# The Emerging Role of Glucagon-like Peptide 1 (GLP-1)-Based Medications in the Treatment of Heart Failure, with a Focus on Heart Failure with Preserved or Mildly Reduced Ejection Fraction

**DOI:** 10.3390/medicina62050870

**Published:** 2026-05-01

**Authors:** Rachel Su Min Lee, Jas-mine Seah, Sara Baqar, Piyush M. Srivastava, Eylem Levelt, Angeline S. Leet, Andrew I. MacIsaac, Elif I. Ekinci, Richard J. MacIsaac

**Affiliations:** 1 Department of Endocrinology, Austin Health, Heidelberg, Melbourne, VIC 3084, Australia; rachelsm@doctors.org.uk (R.S.M.L.);; 2 Department of Medicine, Austin Health, University of Melbourne, Heidelberg, Melbourne, VIC 3084, Australia; p.srivastava@unimelb.edu.au; 3 Department of Cardiology, Austin Health, Heidelberg, Melbourne, VIC 3084, Australia; 4 Advara Heart Care, Mulgrave Private Hospital, Mulgrave, Melbourne, VIC 3170, Australia; 5 Department of Cardiometabolic Imaging, Baker Heart and Diabetes Institute, University of Melbourne, Melbourne, VIC 3004, Australia; 6 Department of Cardiology, St Vincent’s Hospital Melbourne, Fitzroy, Melbourne, VIC 3065, Australia; 7 Department of Medicine, St Vincent’s Hospital Melbourne, University of Melbourne, Fitzroy, Melbourne, VIC 3065, Australia; 8 Australian Centre for Accelerating Diabetes Innovations, School of Medicine, University of Melbourne, Parkville, Melbourne, VIC 3052, Australia; 9 Department of Endocrinology & Diabetes, St Vincent’s Hospital Melbourne, Fitzroy, Melbourne, VIC 3065, Australia

**Keywords:** heart failure, obesity, diabetes, cardiovascular disease, GLP-1 receptor agonists

## Abstract

Glucagon-like Peptide 1 (GLP-1)-based medications have been extensively studied for the management of type 2 diabetes, obesity, chronic kidney disease, and atherosclerotic cardiovascular disease. More recently, their potential role in preventing and treating heart failure has gained increasing attention. Given the strong pathophysiological links among diabetes, obesity, and heart failure, GLP-1-based medications represent a promising therapeutic option to improve morbidity and mortality across these interconnected conditions. In this review, we summarise and discuss recent studies involving GLP-1-based medications that have reported on HF-related outcomes. There is increasing evidence that these medications have beneficial effects on HF outcomes in patients with heart failure with preserved ejection fraction and possibly in those with mildly reduced ejection fraction. The usefulness of GLP-1-based medications in reduced ejection fraction HF remains to be defined.

## 1. Introduction

Glucagon-like Peptide 1 (GLP-1) is a member of the incretin hormone family, which includes gastrointestinal hormones that stimulate post-prandial insulin secretion [1]. Beyond its glucose-lowering properties, agonism of the GLP-1 receptor regulates appetite and promotes weight loss. These agents are well-established in the treatment of type 2 diabetes mellitus (T2DM) and obesity, but they are now also emerging as therapeutic options for the management of other conditions such as obstructive sleep apnoea, metabolic dysfunction-associated steatohepatitis (MASH), chronic kidney disease (CKD), atherosclerotic cardiovascular disease (ASCVD), and heart failure (HF) [2,3].

Recently, novel dual- and triple-receptor incretin agonists have been developed. These agonists combine GLP-1 with hormones such as glucose-dependent insulinotropic polypeptide (GIP) and glucagon (GCG). Synergistically, in addition to GLP-1’s ability to improve glycaemic control and increase satiety, GIP potentiates the incretin effect and mitigates the diabetogenic effects of chronic glucagon receptor agonism, as discussed below [4]. This dual-receptor agonist effect has been used to develop medications such as tirzepatide. Acute stimulation of the glucagon receptor causes hyperglycaemia and is a well-recognised treatment for severe hypoglycaemia. However, chronic glucagon receptor agonism increases energy expenditure and aids weight loss. Survodutide and mazdutide are examples of dual GLP-1 and glucagon agonists. Triple GLP-1/GIP/GCG agonists are also in development; an example of this class of medication is retatrutide. Other GLP-1-based medications that incorporate agonism of the amylin receptor (such as cagrilintide and amycretin, also referred to as zenagamtide) are also undergoing clinical studies to gauge their effectiveness in improving metabolic control, weight, and cardiovascular (CV) outcomes. A summary of the different classes of GLP-1-based medications is shown in Table 1.

In this review, we examine the emerging evidence for the use of GLP-1-based medications in HF. We focus on outcomes in patients with obesity-related heart failure with or without diabetes who have preserved (HFpEF) or mildly reduced ejection fraction (HFmrEF). Table 2 summarises the major GLP-1-based medication trials that report HF outcomes as primary or secondary endpoints, grouped by GLP-1-based medication class. We also discuss the limitations of existing data and the implications for clinical practice and future research for GLP-1-based medications in the setting of HF management.

A structured literature search was conducted across MEDLINE (via PubMed) and Embase from database inception to December 2025. The search strategy incorporated combinations of keywords, including GLP-1 receptor agonist (GLP-1 RA), semaglutide, liraglutide, tirzepatide, dual GLP-1 agonist, heart failure, HFpEF, HFmrEF, Heart failure with reduced ejection fraction (HFrEF), type 2 diabetes mellitus, overweight and obesity, and cardiovascular outcomes.

Eligible studies included randomised controlled trials, prespecified subgroup analyses, mechanistic studies and meta-analyses evaluating GLP-1 receptor agonists with reported cardiovascular- or heart failure-related outcomes. Case reports, non-English publications, and studies without relevant heart failure endpoints were excluded. Titles and abstracts were screened for relevance, followed by a full-text review of eligible articles. Studies were selected based on relevance to HF outcomes and trial design. Reference lists of major trials and reviews were also screened for additional relevant publications.

Data extraction focused on study design, population characteristics, intervention and dosing, and key CV or heart failure outcomes, including hospitalisation for heart failure (HHF) and patient-reported outcomes. Studies were grouped by population (T2DM with a high CV or kidney failure risk, obesity, and specific HFpEF/HFrEF). They were also grouped by endpoint type (HHF and HF well-being scores). Subsequently, data on study design, patient characteristics, interventions, and key HF-related outcomes were extracted. This methodology served as the basis for this review.

To ensure methodological consistency, we defined the primary search window up to December 2025. Given the rapidly evolving nature of studies in this field and to keep our review as contemporary as possible, we also included a few selected high-impact studies published online in early 2026 in our review [5,6,7,8,9,10,11].

## 2. Outcome Trials Reporting HF Events as Secondary Outcomes

The evidence presented in this section includes results from cardiovascular outcome trials (CVOTs) and one kidney outcome trial. We focus on trials reporting HF secondary outcomes, whose results, when interpreted in the context of the trial design, set the scene for dedicated HF outcome trials. As HF-related events were not the primary endpoints of these trials, which were also not conducted in populations exclusively with HF, the results should be interpreted only as hypothesis-generating.

To date, there have been nine dedicated GLP-1 RAs, one placebo-controlled CVOT and one kidney-specific trial (Effects of Semaglutide on Chronic Kidney Disease in Patients with Type 2 Diabetes, FLOW) for people with T2DM that have reported HF events as secondary outcomes. Overall, GLP-1-based medications are associated with a numerical reduction in HF events among patients with T2DM, although only three of 10 trials showed nominal statistical significance for a reduction in HF events (Table 3).

In addition to the limitations in interpreting HF outcomes from these trials, it should be noted that the prevalence of pre-existing HF in the population studies ranged from 7.0% to 23.6% and that definitions of HF and measurements of left ventricular ejection fraction (LVEF) were inconsistently reported. Many trials also did not control for background use of Sodium–Glucose Cotransporter 2 (SGLT2) inhibitors. Importantly, baseline SGLT2 inhibitor use was low (9.6–25.0%), and the implications of this on the impact of GLP-1-based medications in the treatment of HF in clinical practice are discussed later. Outpatient worsening HF events that did not require hospitalisation were not assessed in the above trials.

Regardless of the above, a systematic review and meta-analysis involving 52,752 participants in the Liraglutide and Cardiovascular Outcomes in Type 2 Diabetes (LEADER), Effects of Once-Weekly Exenatide on Cardiovascular Outcomes in Type 2 Diabetes (EXSCEL), Albiglutde and Cardiovascular Outcomes in Patients with Type 2 Diabetes (HARMONY Outcomes), Dulaglutide and Cardiovascular Outcomes in Type 2 Diabetes (REWIND), Semaglutide Effects on Cardiovascular Outcomes in People with Overweight or Obesity (SELECT) and FLOW trials involving participants with T2DM or obesity without known HF showed that treatment with a GLP-1 RA reduced the risk of new-onset HF compared to the placebo (HR: 0.77, 95% CI: 0.65–0.93, *p* < 0.001) and the composite of HF events or CV death (HR: 0.82, 95% CI: 0.76–0.89, *p* < 0.001). The effect of a GLP-1 RA on HF events was independent of its effects on glycated haemoglobin (HbA1c) or weight loss, but it correlated with its protective effects on MACE [23].

## 3. Specific HF Trials in the Setting of Preserved or Mildly Reduced Ejection Fraction

### 3.1. HF Wellbeing Score and Physical Limitations

Recent dedicated HF trials have demonstrated a positive impact of GLP-1-based medications on health status and physical function in patients with obesity-related HFpEF or HFmrEF. However, the interpretation of these findings in HFmrEF should be undertaken with caution. The trials discussed below applied LVEF inclusion thresholds (≥45%) that largely encompass HFpEF or the borderline HFmrEF-HFpEF spectrum, with limited representation of patients with clearly defined HFmrEF (LVEF 41–49%). As such, any observed benefits that specifically apply to HFmrEF need to be interpreted with extreme caution.

The Semaglutide in Patients with Heart Failure with Preserved Ejection Fraction and Obesity (STEP-HFpEF), a trial involving people without diabetes; STEP-HFpEF DM, a trial involving people with diabetes; and Tirzepatide for Heart Failure with Preserved Ejection Fraction and Obesity (SUMMIT) trials, a study involving people with and without diabetes, were the first placebo-controlled randomised studies to investigate the effects of GLP-1-based medications in study populations that specifically enrolled patients with obesity-related HFpEF or HFmrEF (Table 4). The STEP-HFpEF studies evaluated the usefulness of semaglutide (2.4 mg per week), whilst the SUMMIT trial used tirzepatide (up to 15 mg per week) in the above settings [24,25,26].

In the two STEP-HFpEF trials (n = 1145), semaglutide significantly improved the Kansas City Cardiomyopathy Questionnaire Clinical Summary Score (KCCQ-CSS) (primary endpoint) and the 6 min walk distance (6MWD) test at 52 weeks (Table 4) [24,25]. These benefits were consistent across subgroups defined by age, race, gender, systolic blood pressure, body mass index (BMI), baseline C-reactive protein and the range of LVEF for participants included in the studies (LVEF ≥ 45%) [27].

### 3.2. Outcomes Related to Hospitalisation for Heart Failure (HHF)

The STEP-HFpEF and SUMMIT trials also reported clinical events related to HF (Table 5). In the STEP-HFpEF trials, HF events were exploratory endpoints, and the trials were not adequately powered to assess HF outcomes. Although results suggested favourable trends toward reduced clinical HF events, these findings need to be interpreted in the context of very low event numbers and the fact that they were not the primary outcome of interest in the above trials.

In a pooled analysis of STEP-HFpEF and STEP-HFpEF-DM trials involving 573 semaglutide-treated participants and 572 placebo-treated participants, HF events (adjudicated HHF or urgent hospital visit for intravenous therapy) occurred in 1% of the semaglutide group and 5% of the placebo group over 52 weeks. The risk of adjudicated CV death was also lower in the semaglutide (n = 2) versus placebo (n = 5) groups (HR: 0.31, 95% CI: 0.15–10.62) [27].

In contrast, the SUMMIT trial was the first study to evaluate HHF as a primary endpoint in an obesity-related HFpEF population. In this trial, 731 patients with HFpEF and BMI > 30 kg/m^2^ were randomised to tirzepatide or the placebo and followed up for 104 weeks. Adjudicated death from cardiovascular causes or a worsening heart failure event (defined as hospitalisation or intensification of intravenous or oral HF therapy) occurred in 36 patients (9.9%) in the tirzepatide group and in 56 patients (15.3%) in the placebo group (HR: 0.62, 95% CI: 0.41–0.95, *p* = 0.026). Worsening heart failure events occurred in 29 patients (8.0%) in the tirzepatide group and in 52 patients (14.2%) in the placebo group (HR: 0.54, 95% CI: 0.34–0.85).

Of note, in a recent meta-analysis of 7282 participants with obesity- or diabetes-related HFpEF enrolled in the various CVOTs and kidney outcome- and HF-specific trials, including the EXSCEL, SELECT, STEP-HFpEF, STEP-HFpEF DM, SUMMIT and FLOW trials, the risks of CV death or worsening HF (HR: 0.74, 95% CI: 0.63–0.88), worsening HF events (HR: 0.62, 95% CI: 0.47–0.81) and all-cause mortality (HR: 0.81, 95% CI: 0.67–0.99) were significantly reduced with GLP-1 RA treatments compared to the placebo. No significant difference was found in CV mortality alone [28].

Current trials have not been powered to detect differences in mortality. While the overall risk of CV death is reduced with GLP-1-based agents, only three trials demonstrated statistically significant benefits. CV deaths directly attributed to HF were not specified in most trials. In the SUMMIT trial, numerically more CV deaths occurred in the tirzepatide group than in the placebo group (n = 8 [2.2%] versus n = 5 [1.4%]) [26]. However, only four were attributed to worsening HF, and deaths of undetermined causes were also categorised as CV deaths.

More recently, a study using US healthcare claims data identified participants with HFpEF who mimicked the eligibility for entry into the STEP-HFpEF DM and SUMMIT trials. HF-related outcomes for participants initiated on semaglutide or tirzepatide were compared with those initiated on the dipeptidyl peptidase-4 (DPP-4) inhibitor sitagliptin. The main outcome of interest was a composite of HHF or all-cause mortality. The use of semaglutide (HR: 0.58, 95% CI: 0.51–0.65) and tirzepatide (HR: 0.42, 95% CI: 0.31–0.57) was associated with a substantially lower risk of the primary endpoint than sitagliptin. The authors of that study concluded that there was no meaningful difference in the reduced risk of HHF or mortality between tirzepatide and semaglutide.

## 4. HF with Reduced Ejection Fraction

Compared with HFpEF, the effects of GLP-1 RAs in HFrEF have not demonstrated clinical benefit and, in fact, have raised safety concerns for the use of this class of medication in the setting of HFrEF (Table 6). The Functional Impact of GLP-1 for Heart Failure Treatment (FIGHT) (n = 300) and Effect of Liraglutide on left ventricular function in stable chronic heart failure patients with and without diabetes (LIVE) (n = 241) trials reported a numerical increase in HF events (HR: 1.30, 95% CI: 0.89–1.88) and a statistically significant increase in serious cardiac events (10% vs. 3%, *p* = 0.04), respectively, with liraglutide versus the placebo [29,30]. Notably, neither study was designed nor underpowered to investigate CV outcomes as primary endpoints, although the mechanisms underlying the observed adverse outcomes remain unclear. The FIGHT trial enrolled a high-risk population recently hospitalised for HFrEF. In the study, there was a numerical increase in HF events in the liraglutide-treated group compared with the placebo group (63 [41%] vs. 50 [34%], respectively), but this difference did not reach statistical significance. In contrast, the serious cardiac events reported in the liraglutide group (10%) were primarily driven by ventricular tachycardia (including one fatal case), atrial fibrillation and acute coronary syndrome, compared to the placebo group (3%). Of note, treatment with liraglutide was associated with an increase of 6 beats per minute in heart rate, a potential contributing factor to this worse outcome. A modest but consistent increase in heart rate (2–4 beats per minute) has previously been observed in the GLP-1 RA CVOTs [22,26,31]. While the clinical significance of this chronotropic effect remains uncertain, it raises concerns, as it is known that an increase in heart rate is associated with adverse outcomes in patients with HFrEF [32]. Another potential limitation of GLP-1 RA use in the setting of HFrEF relates to weight loss, especially in those with HF-related cachexia, where further weight loss associated with the use of GLP-1-based medications may, in fact, result in a worse prognosis [33].

Regarding functional status outcomes reported in the above studies, the FIGHT trial found no significant improvements in health status or physical function as measured by the KCCQ-CSS and 6MWD at 26 weeks [29]. In contrast, the LIVE study demonstrated modest improvement in 6MWD (+24 metres) with liraglutide therapy compared with the placebo at 24 weeks, although the KCCQ-CSS was not assessed [30].

Currently, the role of GLP-1-based medications in the management of people with HFrEF remains undefined, and the use of these classes of medications warrants caution in this population, as suggested by the LIVE and FIGHT studies. The safety and efficacy of GLP-1-based medications in patients with HFrEF for HF-specific outcomes require further investigation.

### Studies Involving NT-proBNP as a Surrogate HF Outcome Marker

N-terminal pro-B-type natriuretic peptide (NT-proBNP) is a widely used diagnostic and prognostic biomarker in the management of clinical HF. According to the 2019 draft guidance from the US Food and Drug Administration (FDA), NT-proBNP may be useful for risk stratification and prognostication; however, its validity as a surrogate endpoint for clinical benefit in HF trials has not been established [34].

The use of GLP-1 RAs has been associated with reductions in NT-proBNP expression, although only two studies have demonstrated statistically significant changes (Table 7). Interpretation across trials is limited by heterogeneity in patient populations, the specific GLP-1-based agent used, and the timing of the NT-proBNP measurement relative to therapy initiation. Moreover, NT-proBNP expression may remain elevated during the convalescent phase of HF and is influenced by multiple confounding factors, including obesity, age and renal function [35]. For instance, as GLP-1 RAs induce weight loss, the expected reduction in NT-proBNP expression may be attenuated, potentially limiting its sensitivity as a biomarker of treatment response in this context [36].

## 5. Mechanistic Studies

The exact mechanisms underlying the improvement in HF outcomes with GLP-1-based medications, especially in HFpEF, are yet to be fully delineated but are most likely a result of both indirect metabolic effects and direct cardiac actions. This section discusses a combination of preclinical, translational, and early-phase clinical studies and should be interpreted as highlighting potential or hypothesis-generating mechanistic pathways that GLP-1-based medications may influence to improve HF outcomes.

Indirect mechanisms associated with GLP-1 RA-based medication use include anti-inflammatory effects, as well as improvements in metabolic, hemodynamic, endothelial, and volume status, but at present, their relative contribution to improved HF outcomes remains incompletely defined. In contrast, weight reduction with GLP-1-based medications is most likely a major mediator of improved outcomes for people with HFpEF/HFmrEF. However, in the STEP-HFpEF studies, the magnitude of improvement in the KCCQ-CSS was similar across the two trials, despite approximately 40% less weight loss with semaglutide in people with diabetes than in those without diabetes. This suggests that at least some of the effects of semaglutide are independent of weight reduction [24,25].

In addition, GLP-1 RAs have been shown to improve endothelial function and vascular homeostasis by enhancing nitric oxide bioavailability, reducing oxidative stress, and suppressing inflammatory pathways. These effects are mediated by multiple signalling mechanisms, including PPARγ activation and suppression of the NOD-like receptor family and the pyrin domain-containing 3 (NLRP3) inflammasome, contributing to reduced endothelial dysfunction and vascular inflammation; however, the extent to which these effects translate into HF benefits remains uncertain [39].

GLP-1 RAs may also improve HF outcomes and symptoms, as well as improve chronic pulmonary congestion, via mechanisms related to fluid balance owing to their natriuretic properties [40]. In two dedicated HF outcome trials, treatment with semaglutide (2.4 mg subcutaneously weekly) was associated with a significant reduction in both loop diuretic use and dosage over the study period [24,25]. However, further studies are required to determine whether the natriuretic effect of GLP-1 RAs contributes meaningfully to improvements in HF outcomes and pulmonary congestion.

The presence of high-risk cardiomyopathy genotypes may further contribute to the heterogeneity of HFmrEF and HFpEF, potentially modifying responses to therapies targeting metabolic and fibroinflammatory pathways [6]. Recent evidence indicates that micro ribonucleic acid (miRNA)-driven regulation of inflammation–fibrosis signalling is central to HFpEF pathophysiology, with key postulated mediators, such as miR-21 and miR-29, modulating myocardial fibrosis and inflammatory cascades. This provides a molecular framework through which anti-inflammatory and anti-fibrotic therapies may exert beneficial effects, although direct modulation of these pathways by GLP-1 RAs has not been clearly established [41].

Other direct mechanistic pathways play an important role in improving outcomes for the above patients. The localisation of GLP-1, GCG, GIP, and amylin receptors in the human heart is primarily detected at the mRNA level, with limited and inconsistent protein-level confirmation, and their functional relevance in cardiac tissue remains incompletely understood. The GLP-1 receptor has been localised to the sinoatrial node of the heart and, potentially, the atrium, though its presence in ventricular myocytes is uncertain [42]. The GCG receptor shows differential expression between the atria and ventricles of the human heart, which is influenced by genetic variation and inter-study differences [43,44]. In contrast, the GIP receptor is expressed in ventricular and atrial cardiomyocytes, as well as adipocytes and pericytes, in the human heart [44]. The expression profile of amylin receptors in the human heart remains inadequately characterised [45]. Direct effects of GLP-1-based medications on the heart most likely include a reduction in epicardial fat, a metabolic shift towards glucose oxidation, improved calcium handling in the myocardium, improved mitochondrial function, and anti-fibrotic effects [7,46].

Of note, and as mentioned previously, GLP-1-based medications increase the heart rate via still poorly understood mechanisms, with most studies reporting an increase of approximately 2–4 beats per min [22,26]. An increase in heart rate in patients with HFpEF may be beneficial and may contribute to the improved outcomes reported for GLP-1 RA-treated patients with HFpEF, as heart rate-reducing medications such as beta blockers may pose a risk of harm in this setting. In contrast, for patients with HFrEF, an increase in heart rate may have deleterious effects, as heart rate-reducing medications have benefits in HFrEF patients [47]. In a recent retrospective observational analysis of patients with HFrEF and implanted cardiac devices, GLP-1 RA use was associated with a significant increase in heart rate (by 7 beats per minute) and an increased number of non-sustained ventricular events and total shock/antitachycardia pacing therapies. The authors of the study suggested that this finding again highlights the need for further evaluation of GLP-1 RA use in HFrEF [32].

The role of myocardial perfusion and energetics as mediators of cardiac outcomes with GLP-1-based medications has been investigated in a mechanistic randomised crossover study comparing liraglutide with pioglitazone treatment in people with T2DM (n = 41). After 16 weeks, liraglutide significantly increased stress myocardial blood flow (MBF) (1.62 mL/g/min to 2.08 mL/g/min; *p* = 0.01), myocardial perfusion reserve (MPR) (2.40 to 2.90; *p* = 0.01), and both resting (1.47 to 1.94, *p* = 0.00002) and stress PCr/ATP ratios (1.32 to 1.58; *p* = 0.004) [48]. These effects were not demonstrated with pioglitazone therapy.

In a Cardiac Magnetic Resonance (CMR) substudy of the SUMMIT trial (n = 106), the tirzepatide group demonstrated reduced paracardiac (epicardial plus pericardial) adipose tissue (−45 mL, 95% CI: −69 to −22, *p* < 0.001) and LV mass, which correlated with weight loss (−11 g, 95% CI: −19 to −4, *p* = 0.004) at 52 weeks [49]. However, the study did not utilise late gadolinium enhancement to detect myocardial fibrosis or distinguish between cardiomyocyte and extracellular volume; it is unknown whether LV mass reductions reflected decreased cardiomyocyte mass, reduced interstitial fibrosis, or both.

Another proposed mechanism by which GLP-1 RAs may improve outcomes in HFpEF patients is by reducing Epicardial Adipose Tissue (EAT) thickness [50,51]. In overweight individuals with diabetes, liraglutide was associated with a 36% reduction in EAT over six months, compared to no significant change with metformin [51]. Due to its proximity to the myocardium, EAT may exert adverse mechanical effects on both systolic and diastolic cardiac function. Additionally, excess fatty acids released from EAT can be taken up by cardiomyocytes, further contributing to cellular dysfunction and apoptosis [50]. Notably, clinical data indicate that increased pericardial fat volume—a combination of epicardial and paracardial fat—is associated with a higher risk of HF, particularly HFpEF, even in individuals without pre-existing CVD (n = 6785) [52].

Recently, a human genetic study based on cis-Mendelian randomisation (which uses naturally occurring genetic variants located within or near a gene coding for a drug target) showed that genetically proxied GLP-1 receptor activation (using HbA1c as a surrogate marker of activation) is associated with approximately a 50% lower risk of heart failure. The HF outcome was derived from a genome-wide association meta-analysis comprising seven original studies and 1,665,481 participants. Of note, the association between GLP-1 receptor activation and heart failure risk persisted after adjustment for BMI and T2DM. The authors of the above study suggested that their findings supported evaluating the effects of GLP-1RAs in non-obese, non-diabetic HF patients [8].

### GLP-1 RA: Tolerability and Adverse Events

A summary of adverse events from the GLP-1-based medication studies that have been reported on HF-related outcomes and events is shown in Table 8. As expected, given the mode of action of GLP-1-based medications, gastrointestinal adverse events, including nausea, vomiting, constipation or diarrhoea, are the most common side effects, and although typically transient, these side effects necessitate discontinuation in some patients. In contrast, several less common and less well-established adverse events associated with GLP-1-based medications include biliary disease and pancreatitis; however, definitive causal links have not been established for these outcomes. Additional emerging or debated adverse associations include suicidal ideation, exacerbation of diabetic retinopathy, development of non-arteritic optic neuropathy and excessive mass loss. However, once again, causality for the above risks with GLP-1 RAs use has yet to be established. Severe hypoglycaemia with GLP-1 RAs, outside of their use in combination with insulin or sulfonylureas, is not a clinical concern [53].

In general, although permanent discontinuation rates due to adverse events are greater in GLP-1 RAs versus the placebo (HR: 1.51, 95% CI: 1.18–1.94), usually no difference or fewer serious adverse events (SAEs) are reported with GLP-1 RA use in CVOTs (HR: 0.95, 95% CI: 0.90–1.01) [2]. Reassuringly, in dedicated GLP-1 RA HF trials performed to date, SAEs have been largely similar between active- (10.0–69.1%) and placebo-treated (3.0–72.5%) participants (Table 8). Beyond safety, other barriers to implementation include drug cost and availability across different healthcare services and systems.

## 6. Ongoing Studies

The Effect of Retatrutide Once Weekly on Cardiovascular Outcomes and Kidney Outcomes in Adults (TRIUMPH-OUTCOMES) trial aims to explore the CV and renal effects of retatrutide, a triple GLP-1/GIP/GCG agonist, in overweight people with pre-existing ASCVD and/or CKD. Primary outcome measures include the time to the first occurrence of composite endpoints, including HF events [55].

Maridebart Cafraglutide (MariTide) in Heart Failure with Preserved or Mildly Reduced Ejection Fraction and Obesity (MARITIME-HF) is another phase III trial that will investigate the effectiveness of MariTide, a GIP RA conjugated to GLP-1 analogues, in reducing complications and mortality in patients with obesity-related HFpEF or HFmrEF.

A CVOT involving amycretin (Zenagamtide), a unimolecular GLP-1 and amylin receptor agonist, is also planned to start in the second half of 2026. The primary endpoint of that trial will be the time to a four-point MACE, including HF hospitalisations.

### Comparison of GLP-1-Based Medications to SGLT2 Inhibitors

Although several trials suggest potential benefits of other agents in HFpEF, only SGLT2 inhibitors are currently recommended as first-line therapy [56]. Finerenone has received FDA approval for HFmrEF or HFpEF (LVEF ≥ 40%) following results from the Finerenone in Heart Failure with Mildly Reduced or Preserved Ejection Fraction (FINEARTS-HF) trial [57]. However, it is not yet incorporated as a class I recommendation in the current American College of Cardiology (ACC) or European Society of Cardiology (ESC) guidelines for HFpEF, which predate these data [56,58].

SGLT2 inhibitors remain the only class with consistent benefits in reducing CV death and HHF across HF phenotypes. These benefits extend to improved quality of life and enhanced diuretic efficiency. In general, event rates have been reduced by 30% in HF trials involving SGLT2 inhibitors [9].

SGLT2 inhibitors now represent foundational therapy in HFpEF. However, the limited background use of SGLT2 inhibitors in both GLP-1 RA CVOTs and dedicated HFpEF trials (including STEP-HFpEF and SUMMIT) is a key limitation for assessing the true impact of GLP-1 RAs in reducing major CV and HF outcomes in everyday clinical practice. At present, the incremental benefit of GLP-1 RA therapy on top of background SGLT2 inhibitor use remains uncertain and represents an important area for future investigation.

In a pooled analysis of four trials involving 3743 participants with HFpEF and the GLP-1 RA semaglutide (SELECT, FLOW, STEP-HFpEF and STEP-HFpEF DM), semaglutide reduced the risk of the composite HHF/CV death endpoint by 31% compared to the placebo (HR: 0.69, 95% CI: 0.53–0.89, *p* = 0.0045) [59]. Worsening HF events were also significantly reduced (2.8% versus 4.7%; HR: 0.59, 95% CI: 0.41–0.82, *p* = 0.0019), with a number needed to treat (NNT) of 97 to prevent one composite endpoint event and an NNT of 95 for one worsening HF event over one year. These findings should be interpreted in comparison to those reported for SGLT2 inhibitors, which have demonstrated NNTs of only 30–32 for the composite HHF/CV death endpoint [60]. While direct comparisons across trials should be made with caution, the above findings suggest a greater magnitude of benefit with SGLT2 inhibitors compared to GLP-1 RAs in patients with HFpEF.

## 7. Conclusions

A declining trend in hospitalisation of HF among people with diabetes is already evident, and this decrease would be anticipated to continue given the development of new HF therapies [61]. There is now a growing body of evidence supporting the role of GLP-1-based medications to improve HF-related symptoms, physical limitations, and prognosis in people with obesity-related HFpEF, with or without diabetes. In contrast, the risk-benefit of GLP-1-based medications in people with HFrEF remains unclear and requires further study.

The potential benefit of GLP-1 receptor agonists as HF medications has recently been highlighted by a recent large real-world study. This particular study suggested that the use of GLP-1 RAs is associated with a lower risk of hospitalisation for HF than DPP-4 inhibitors, and that the risk reduction was similar to that observed among users of SGLT2 inhibitors. This effect was seen in patients with and without established heart failure [10].

Interestingly, this finding contrasts with the generally greater benefits of SGLT2 inhibitors for HF outcomes reported in clinical trials, as mentioned above.

When comparing different classes of GLP-1-based medications for HF events, semaglutide has been shown to improve symptoms, physical limitations, exercise function, and quality of life in obese patients with HFpEF (with or without diabetes) and to reduce the risk of worsening HF events. Although results similar to those above have been reported for tirzepatide, in the SUMMIT study, participants treated with tirzepatide showed a slight increase in total mortality compared with placebo-treated patients. The US FDA has indicated that a treatment for heart failure may be approvable based solely on effects on symptoms and physical function [34]. It is our understanding that an HFpEF indication for semaglutide is being pursued, but not for tirzepatide at this point. Indeed, the European Medicines Agency’s Committee for Medicinal Products for Human Use has recommended that the product information for tirzepatide be updated to include data from the SUMMIT study, which demonstrated its positive effects in people with symptomatic chronic HFpEF [11]. Larger and longer trials specifically focusing on HF-related clinical events after GLP-1-based medication use will possibly be required by regulatory bodies before an indication for GLP-1-based medications as a treatment option for HFpEF is granted.

Where GLP-1-based medications will eventually fit into the paradigm of HFpEF management remains to be established. We also await the results of further mechanistic studies to help delineate whether the benefits of GLP-1-based medications on HF outcomes are attributable solely to weight loss or to other distinct mechanisms. In addition, the usefulness of GLP-1-based medications in HFrEF remains to be defined.

## Figures and Tables

**Table 1:** Examples of GLP-1-based medications on the market or in development.

Type	Drug/Platform	Route/Schedule	Status in the USA (2026)	Status in Australia (2026)
GLP-1 RA	Albiglutide (Tanzeum and Eperzan)	SC weekly	Discontinued	Discontinued
	Dulaglutide (Trulicity)	SC weekly	Marketed	Marketed
	Efpeglenatide	SC weekly	Not marketed (late-stage development/regulatory activity)	Not marketed
	Exenatide (Byetta/Bydureon)	SC; twice daily (Byetta) or weekly (Bydureon)	Bydureon marketed; Byetta discontinued	Discontinued
	Liraglutide (Victoza and Saxenda)	SC daily	Marketed	Marketed
	Lixisenatide (Adlyxin)	SC daily	Discontinued	Discontinued
	Subcutaneous semaglutide (Ozempic and Wegovy)	SC weekly	Marketed	Marketed
	Oral semaglutide (Rybelsus)	Oral daily	Marketed	Marketed
	High-dose oral semaglutide “Wegovy pill”	Oral daily	Marketed	Not approved/ not marketed
Non-peptide oral GLP-1 RA	Orforglipron (Foundayo)	Oral daily	Marketed (FDA approved 2026)	Under evaluation (not marketed)
Dual GLP-1/GIP receptor agonist	Tirzepatide (Mounjaro and Zepbound)	SC weekly	Marketed	Marketed (Mounjaro only)
Dual GLP-1/GIP receptor antagonist	MariTide (Maridebart Cafraglutide)	SC monthly	Phase 3 (not marketed)	Not marketed
Dual GLP-1/GCG receptor agonists	Survodutide + Mazdutide	SC weekly	In the late phase of development	Not marketed
GLP-1 RA + Amylin analogue combination	Cagrilintide + Semaglutide (CagriSema)	SC weekly	Phase 3	Not marketed
Unimolecular GLP-1 + Amylin receptor agonist	Amycretin (Zenagamtide)	SC weekly/ Oral daily	SC formulation in late-phase development; oral formulation in early phase development	Not marketed
Triple GLP-1/GIP/GCG agonist	Retatrutide	SC weekly	Phase 3	Not marketed

**Table 2:** Summary of trials using GLP-1-based medications that have reported on HF-related events and outcomes as primary or secondary endpoints.

Agent	Clinical Trial	Dose/Route	Population	Total Participants (Active/Placebo)	Cardiovascular and Heart Failure-Related Outcomes Reported
Albiglutide	HARMONY Outcomes	Up to 50 mg SC weekly (protocol-directed uptitration)	≥40 years with T2DM and established ASCVD	9463 (4728/4735)	↓ 3-point MACE (HR 0.78); neutral on HF hospitalisation.
Dulaglutide	REWIND	1.5 mg SC weekly	≥50 years with T2DM and CV risk factors	9901 (4949/4952)	↓ 3-point MACE (HR 0.88); ↓ HF hospitalisation (HR 0.88)
Exenatide QW	EXSCEL	2.0 mg SC weekly	T2DM ± established CVD	14,752 (7370/7382)	Neutral 3-point MACE (HR 0.91); neutral on HF hospitalisation
Liraglutide	LEADER	1.8 mg SC daily	T2DM at high CV risk	9340 (4661/4679)	↓ 3-point MACE (HR 0.87); ↓ CV death (HR 0.78); no significant reduction in HF hospitalisation
	FIGHT	1.8 mg SC daily (target dose)	HFrEF (LVEF ≤ 40%), recent (within 14 days) hospitalisation and on oral diuretic	300 (154/146)	Numerically, more HF rehospitalisation in the Liraglutide arm
	LIVE	1.8 mg SC daily (target dose)	Clinically stable HFrEF (LVEF ≤ 45%) and on optimal HF treatment	241 (122/119)	Neutral on LV function/death; ↑ serious cardiac events and arrhythmias
Lixisenatide	ELIXA	20 μg SC daily (target dose)	T2DM with recent acute coronary syndrome (MI or unstable angina)	6068 (3034/3034)	Neutral 4-point MACE (HR 1.02); neutral on HF hospitalisation
Semaglutide	FLOW (substudy)	1.0 mg SC weekly (after dose escalation)	T2DM with CKD	3533 (1767/1766)	↓ HF events in HFpEF substudy (HR 0.79); ↓ MACE (HR 0.78)
	PIONEER 6	14 mg oral daily (after dose escalation)	T2 DM and high CV risk or established CVD/CKD	3183 (1591/1592)	Noninferior MACE (HR 0.79); ↓ CV death (HR 0.49); neutral on HF hospitalisation
	SOUL	14 mg oral daily (after dose escalation)	T2DM and established CVD or CKD	9650 (4825/4825)	↓ MACE (HR 0.86); ↓ HF hospitalisation (HR 0.87); ↓ CV death
	SUSTAIN-6	0.5 mg or 1.0 mg SC weekly	T2DM at high CV risk	3297 (1648/1649)	↓ 3-point MACE (HR 0.74); neutral on HF hospitalisation
	SELECT	2.4 mg SC weekly (target dose)	Established CVD with overweight/obesity, without diabetes	17,604 (8803/8801)	↓ MACE (HR 0.80); ↓ HF events (HR 0.75); ↓ atherosclerotic events
	STEP-HFpEF	2.4 mg SC weekly (target dose)	HFpEF (LVEF ≥ 45%) and obesity, without diabetes	529 (264/265)	↓ KCCQ symptoms (+7.8-point improvement); ↓ worsening HF; NT-proBNP reduction
	STEP-HFpEF DM	2.4 mg SC weekly (target dose)	HFpEF (LVEF ≥ 45%), with obesity and diabetes	616 (310/306)	↓ KCCQ symptoms (+7.3-point improvement); hierarchical composite favouring semaglutide (win ratio 1.41–1.73); ↓ HF events; consistent across HbA1c
Tirzepatide	SUMMIT	5–15 mg SC weekly (titrated)	HFpEF (LVEF ≥ 50%) and obesity, with and without diabetes	731 (364/367)	↓ KCCQ symptoms (+6.9-point improvement); ↓ worsening HF (HR 0.54)

Manufacturers: Albiglutide (Tanzeum/Eperzan; GlaxoSmithKline, Brentford, UK); Dulaglutide (Trulicity; Eli Lilly, Indianapolis, IN, USA); Exenatide (Bydureon; AstraZeneca, Cambridge, UK); Liraglutide (Victoza/Saxenda; Novo Nordisk, Bagsværd, Denmark); Lixisenatide (Adlyxin; Sanofi, Paris, France); Semaglutide (Ozempic/Wegovy/Rybelsus; Novo Nordisk, Bagsværd, Denmark); Tirzepatide (Mounjaro/Zepbound; Eli Lilly, Indianapolis, IN, USA).

**Table 3:** Heart failure outcomes from CVOTs or kidney outcome trials involving GLP-1-based medications.

Clinical Trial	Agent/ Dose	n=	Pre-Existing CVD	Pre-Existing HF	Median Follow-Up (Weeks)	HHF Hazard Ratio (95% CI)	CV Death Hazard Ratio (95% CI)
ELIXA (2015) [12]	Lixisenatide 20 mcg daily, SC (target dose)	6068	100.0%	22.5%	109	0.96 (0.75–1.23) *p* = 0.75	0.98 (0.78–1.22) *p* = 0.85
LEADER (2016) [13]	Liraglutide 1.8 mg daily, SC	9340	81.0%	18.0%	198	0.87 (0.73–1.05) *p* = 0.14	0.78 (0.66–0.93) *p* = 0.007
SUSTAIN-6 (2016) [14]	Semaglutide 0.5 mg or 1.0 mg weekly, SC	3297	83.0%	23.6%	109	1.11 (0.77–1.61) *p* = 0.57	0.98 (0.65–1.48) *p* = 0.92
EXSCEL (2017) [15]	Exenatide 2 mg weekly SC	14,752	73.0%	16.0%	167	0.94 (0.78–1.13) *p* = 0.49	0.88 (0.76–1.02) *p* = 0.63
Harmony Outcomes (2018) [16]	Albiglutide up to 50 mg weekly, SC (protocol directed uptitration)	9463	100%	20.2%	83	0.71 (0.53–0.94)	0.93 (0.73–1.19) *p* = 0.58)
PIONEER 6 (2019) [17]	Semaglutide 14 mg daily, oral (after dose titration)	3183	84.7%	12.2%	68	0.86 (0.48–1.55)	0.49 (0.27–0.92)
REWIND (2019) [18]	Dulaglutide 1.5 mg weekly, SC	9901	31.5%	8.5%	282	0.93 (0.77–1.12) *p* = 0.46	0.91 (0.78–1.06) *p* = 0.21
AMPLITUDE-O (2021) [19]	Efpeglenatide 4 mg or 6 mg weekly, SC	4076	89.6%	18.0%	94	HF events: 0.61 (0.38–0.98)	0.72 (0.5–1.03)
FLOW substudy (2024) [20]	Semaglutide 1 mg weekly, SC (after dose escalation)	3533	23.0%	19.2%	177	HF events: 0.74 (0.58–0.94) *p* = 0.015	0.71 (0.66–0.94)
SOUL (2025) [21]	Semaglutide 14 mg daily, oral (after dose escalation)	9650	70.7%	23.1%	215	0.90 * (0.79–1.03)	0.93 (0.80–1.09)
SELECT (2023) [22]	Semaglutide 2.4 mg weekly, SC (target dose)	17,604	100.0%	25.0%	173	0.79 (0.60–1.03)	0.85 (0.71–1.01), *p* = 0.07

* A secondary analysis from SOUL has shown that in participants with T2DM, atherosclerotic CV disease and/or CKD and a history of HF at baseline, the HR for the risk of a HF outcome (HF hospitalisation, urgent HF visit, or CV death) was 0.78 (95% CI: 0.63–0.96)—a reduction driven entirely by those with HFpEF [5].

**Table 4:** Trials of GLP-1-based medications involving populations with HFpEF/HFmEF reporting HF well-being scores and physical limitations.

Clinical Trial	Population	Sample Size (n=)	Agent/Maximum Tolerated Dose	Median Follow-Up (Weeks)	Mean Change in KCCQ-CSS (Points)	Mean Change in 6MWD (m)
STEP-HFpEF (2023) [24]	HFpEF (LVEF ≥ 45%), BMI ≥ 30 with no diabetes	529	Semaglutide 2.4 mg weekly, SC	52	7.8 (4.8–10.9) *p* < 0.001	20.3 (8.6–32.1) *p* < 0.001
STEP-HFpEF DM (2024) [25]	HFpEF (LVEF ≥ 45%), BMI ≥ 30 and T2DM	616	Semaglutide 2.4 mg weekly, SC	57	7.3 (4.1–10.4) *p* < 0.001	14.3 (3.7–24.9) *p* = 0.008
SUMMIT (2024) [26]	HFpEF (LVEF ≥ 50%) and BMI ≥ 30	731	Tirzepatide 15 mg weekly, SC	104	6.9 (3.3–10.6) *p* < 0.001	18.3 (9.9–26.7) *p* < 0.001

**Table 5:** Trials of GLP-1-based medications involving populations with HFpEF/HFmEF reporting clinical HF events and/or CV death.

Clinical Trial	Population	Agent/Maximal Tolerated Dose	n=	Median Follow-Up (Weeks)	HF Events Hazard Ratio (95% CI)	CV Death (Number (n)) or Hazard Ratio (95% CI)	Composite HF Events/CV Death Hazard Ratio (95% CI)
STEP-HFpEF (2023)	HFpEF (LVEF ≥ 45%), BMI ≥ 30 with no diabetes	Semaglutide 2.4 mg weekly, SC	529	52	0.08 (0.00–0.42)	Semaglutide = 0 (0%) placebo = 1 (0.4%)	N/A
STEP-HFpEF DM (2024)	HFpEF (LVEF ≥ 45%), BMI ≥ 30 and T2DM	Semaglutide 2.4 mg weekly, SC	616	57	0.40 (0.15–0.92)	Semaglutide = 1 (0.3%) Placebo = 4 (1.3%)	N/A
SUMMIT (2024)	HFpEF (LVEF ≥ 50%) and BMI ≥ 30	Tirzepatide 15 mg weekly, SC	731	104	0.54 (0.34–0.85)	Tirzepatide = 8 (2.2%), Placebo = 5 (1.4%) HR 1.58 (0.52–4.83)	0.62 (0.41–0.95), *p* = 0.026

**Table 6:** GLP-1-based medication trials involving populations with HFrEF.

Clinical Trial	Population	Sample Size (n=)	Agent/Maximum Tolerated Dose	Median Follow-Up (Weeks)	Mean Change in KCCQ-CSS (Points)	Mean Change in 6MWD (m)
FIGHT (2016) [29]	HFrEF (LVEF ≤ 40%), recent (within 14 days) hospitalisation and on oral diuretic	300	Liraglutide 1.8 mg daily, SC	26	0.6 (−4.5–5.8), *p* = 0.81	5 (−29–39) *p* = 0.79
LIVE (2017) [30]	Clinically stable HFrEF (LVEF ≤ 45%) and on optimal HF treatment	241	Liraglutide 1.8 mg daily, SC	24	N/A	24 (2–47) *p* = 0.04

**Table 7:** GLP-1-based medication trials investigating changes in NT-proBNP.

Agent	Clinical Trial	NT-proBNP Endpoint	NT-proBNP
Liraglutide	Avogaro et al. [37]	Change from baseline (SD units)	−0.14 SD (−0.27 to −0.01), *p* = 0.03
	Liu et al. [38]	Weighted Mean Difference between GLP-1 RA versus control	WMD −20.02 pg/mL, *p* = 0.24
	FIGHT [29]	Time-averaged proportional change (0–180 day)	−155 pg/mL (−1368–1058), *p* = 0.80
	LIVE [30]	Mean difference between GLP-1 RA and placebo at 24 weeks	−140 pg/mL (−317–37), *p* = 0.12
Semaglutide	STEP-HFpEF substudy [36]	Treatment ratio at 52 weeks	Treatment ratio 0.82 (0.74–0.91), *p* = 0.0002
Tirzepatide	SUMMIT [26]	Treatment ratio at 52 weeks	Ratio of geometric means = 0.90 (0.79–1.01)

**Table 8:** Adverse events leading to treatment discontinuation and serious adverse events with GLP-1-based medications compared to the placebo in studies that report HF outcomes.

Agent	Clinical Trial	Dose/Route	Total (Active/Placebo Participants	Adverse Events Leading to Treatment Discontinuation (Active/Placebo)	Serious Adverse Events (Active/Placebo)
Semaglutide	SUSTAIN-6 [14]	0.5 mg or 1.0 mg weekly, SC	1648/1649	119 (14.5%)/ 63 (7.6%)	276 (33.6%)/ 298 (36.1%)
	FLOW [20]	1.0 mg weekly, SC	1767/1766	233 (13.2%)/ 211 (11.9%)	877 (49.7%)/ 950 (53.8%)
	SELECT [22]	2.4 mg weekly, SC	8803/8801	1461 (16.6%)/ 718 (8.2%)	2941 (33.4%)/ 3204 (36.4%)
	PIONEER 6 [17]	14 mg daily, oral	1591/1592	184 (11.6%)/ 26 (1.6%)	301 (18.9%)/ 358 (22.5%)
	STEP-HFpEF [24]	2.4 mg weekly, SC	263/266	35 (13.3%)/ 14 (5.3%)	35 (13.3%)/ 71 (26.7%)
	STEP-HFpEF DM [25]	2.4 mg weekly, SC	310/306	33 (10.6%)/ 25 (8.2%)	55 (17.7%)/ 88 (28.8%)
	SOUL [21]	14 mg daily, oral	4825/4825	749 (15.5%)/ 559 (11.6%)	2312 (47.9%)/ 2427 (50.3%) *p* = 0.02
Lixisenatide	ELIXA [12]	20 µg daily, SC	3034/3034	347 (11.4%)/ 217 (7.2%)	669 (22.1%)/ 625 (20.6%)
Liraglutide	LEADER [13]	1.8 mg daily, SC	4668/4672	444 (9.5%)/ 339 (7.3%), *p* < 0.001	2320 (49.7%)/ 2354 (50.4%) *p* = 0.51
	FIGHT [29]	1.8 mg daily, SC	154/146	19.0%/17.0%	98 (63.6%)/ 71 (48.6%)
	LIVE [42]	1.8 mg daily, SC	122/119	11 (9.0%)/ 4 (3.7%)	12 (10.0%)/ 3 (3.0%), *p* = 0.04
Exenatide	EXSCEL [15]	2.0 mg weekly SC	7356/7396	705 (9.6%)/ 492 (6.7%)	1234 (16.8%)/ 1222 (16.6%)
Albiglutide	Harmony Outcomes [16]	50 mg weekly SC	4731/4732	409 (9.0%)/ 307 (6.0%)	1497 (13.7%)/ 1678 (35.6%)
Dulaglutide	REWIND [18]	1.5 mg weekly, SC	4949/4952	451 (9.1%)/ 310 (6.3%)	3424 (69.1%)/ 3592 (72.5%)
Efpeglenatide	AMPLITUDE-O [19]	4.0 mg or 6.0 mg weekly, SC	2717/1359	147 (5.4%)/ 49 (3.6), *p* = 0.02	335 (12.3%)/ 164 (12.1%)
Tirzepatide	SURPASS-4 [54]	5 mg, 10 mg or 15 mg weekly, SC	997/1005	36 (11.0%)/ 54 (5.0%)	41 (12.0%)/ 193 (19.0%)
	SUMMIT [26]	5–15 mg weekly, SC	364/367	23 (6.3%)/ 5 (1.4%)	96 (26.4%)/ 94 (25.6%)

## Data Availability

No new data were created or analysed in this study.
